# Blocking autophagy enhanced cytotoxicity induced by recombinant human arginase in triple-negative breast cancer cells

**DOI:** 10.1038/cddis.2014.503

**Published:** 2014-12-11

**Authors:** Z Wang, X Shi, Y Li, J Fan, X Zeng, Z Xian, Z Wang, Y Sun, S Wang, P Song, S Zhao, H Hu, D Ju

**Affiliations:** 1 Department of Biosynthesis and Key Lab of Smart Drug Delivery of MOE, School of Pharmacy, Fudan University, Shanghai, China; 2 Department of Biopharmaceutical Research, Shanghai Institute of Pharmaceutical Industry, Shanghai, China; 3 Department of Pulmonary Medicine, People's Affiliated Hospital of Fujian University of Traditional Chinese Medicine, Fuzhou, Fujian, China; 4 Department of Head and Neck Surgery, Changzheng Hospital, Second Military Medical University, Shanghai, China

## Abstract

Depletion of arginine by recombinant human arginase (rhArg) has proven to be an effective cancer therapeutic approach for a variety of malignant tumors. Triple-negative breast cancers (TNBCs) lack of specific therapeutic targets, resulting in poor prognosis and limited therapeutic efficacy. To explore new therapeutic approaches for TNBC we studied the cytotoxicity of rhArg in five TNBC cells. We found that rhArg could inhibit cell growth in these five TNBC cells. Intriguingly, accumulation of autophagosomes and autophagic flux was observed in rhArg-treated MDA-MB-231 cells. Inhibition of autophagy by chloroquine (CQ), 3-methyladenine (3-MA) and siRNA targeting Beclin1 significantly enhanced rhArg-induced cytotoxic effect, indicating the cytoprotective role of autophagy in rhArg-induced cell death. In addition, *N*-acetyl-l-cysteine (NAC), a common antioxidant, blocked autophagy induced by rhArg, suggesting that reactive oxygen species (ROS) had an essential role in the cytotoxicity of rhArg. This study provides new insights into the molecular mechanism of autophagy involved in rhArg-induced cytotoxicity in TNBC cells. Meanwhile, our results revealed that rhArg, either alone or in combination with autophagic inhibitors, might be a potential novel therapy for the treatment of TNBC.

Breast cancer, the most common cause of cancer death in women, is a kind of complex and heterogeneous neoplasm.^1^ Approximately 15% of breast carcinomas are triple-negative breast cancers (TNBCs), which have high rates of recurrences and mortality.^2^ TNBCs are defined by the lack of expression of estrogen receptor, progesterone receptor and human epidermal growth factor receptor type 2 (HER2). These tumors are characterized by clinically aggressive behaviors, high recurrence rate and poor prognosis. Owing to lack of targeted therapies (such as hormone therapy or anti-HER2 therapy), currently chemotherapy is the primary treatment for TNBC.^3^ Therefore, investigating new therapeutic approaches is urgently needed for improving the clinical outcome of TNBC therapy.

Recently, deprivation of l-arginine has been a potential therapeutic method for cancers.^4^ By culturing cells in the arginine-free media, a variety of human cancer cells have been found to be auxotrophic for arginine, depletion of which resulted in cell death. Importantly, recombinant human arginase (rhArg) has shown potent anticancer effect in acute myeloid leukemia and acute lymphoblastic T-cell leukemia and solid tumors *in vitro* and *in vivo*^5, 6, 7, 8, 9^ and is currently under clinical investigation for the treatment of melanoma^10^ and hepatocellular carcinoma (HCC).^11^ These carcinomas are auxotrophic for arginine, mainly because of the absence of arginine endogenous synthetical pathway. However, there are no reports about the efficiency in the therapy of breast cancer by rhArg through depletion of arginine.

An increasing number of studies have shown that autophagy is stimulated in response to external stressors (such as starvation and oxidative stress) and internal needs (for example, removal of aggregate-prone proteins).^12^ Autophagy is an evolutionarily conserved catabolic process responsible for the routine degradation of bulk superfluous or dysfunctional proteins and organelles.^13^ Autophagy serves as a protective role in response to a majority of anticancer drugs and in the pathogenesis process.^14, 15^ Not surprisingly, the relationship between autophagy and apoptosis, both genetically regulated and evolutionarily conserved, is complex, and appears to be related to cellular contexts.^16^ Meanwhile, mounting evidence accumulated has revealed that autophagy stimulation and reactive oxygen species (ROS) are closely linked in response to cancer therapeutics.^17, 18^ Notably, the essential contribution of mitochondrially generated ROS in the modulation of autophagy during starvation has been highlighted.

In this study, we investigated whether rhArg might be a potential therapy for TNBC. We reported for the first time that rhArg-induced cell growth inhibition and caspase 3-independent apoptosis in MDA-MB-231 cells. Also, we found that rhArg could induce autophagy in MDA-MB-231 cells in a dose- and time-dependent manner. Interestingly, blocking autophagy potentiated cytotoxicity induced by rhArg, indicating that autophagy had a cytoprotective role in the treatment of rhArg. Meanwhile, ROS was involved in the autophagy and cell growth inhibition induced by rhArg. With our findings mentioned above, rhArg has shown potential to be a promising therapy for TNBC. Furthermore, the combination with autophagy-targeting drugs displayed multipronged treatment for breast cancer therapy.

## Results

### Sensitivity of TNBC to rhArg correlated with sucargininosuccinate synthetase (ASS) and ornithine transcarbamylase (OTC) expression

**Figure 1:**
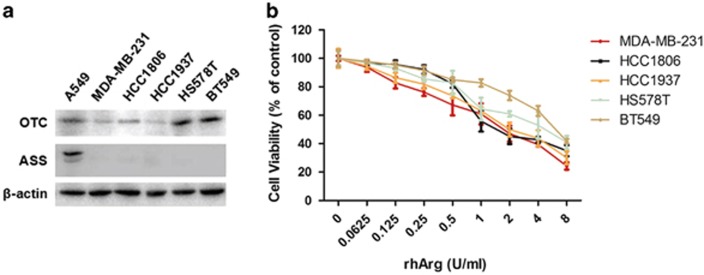
Five TNBC cell lines profiled for ASS, OTC expression and rhArg sensitivity. (**a**) Western blot analysis showed the protein expression of ASS and OTC in A549, MDA-MB-231, HCC-1806, HCC-1937, HS-578T and BT-549 cells. A549 cells were used as positive control. (**b**) MDA-MB-231, HCC-1806, HCC-1937, HS-578T and BT-549 cells were treated with rhArg at indicated concentrations for 3 days. The cell viability was determined by MTT assay at the wavelength of 570 nm (*n*=3, means±S.D.)

ASS and OTC are key enzymes in the synthesis of arginine from citrulline in the urea cycle. ASS and OTC protein levels in five TNBC cell lines (MDA-MB-231, HCC-1806, HCC-1937, HS-578T and BT-549 cells) were evaluated by western blot (Figure 1a). A549 was reported to be resistant to rhArg and its expression of ASS and OTC was used as positive control.^10^ To determine the growth inhibition effect of rhArg on breast cancer cells proliferation *in vitro*, these five breast cancer cells were treated with increasing concentrations of rhArg for 72 h. We observed that all the five TNBC cells were sensitive to rhArg, but rhArg inhibited cell growth in different degree (Figure 1b). These were consistent with the results of ASS and OTC expression. Consequently, these results suggested that sensitivity of TNBC to rhArg is related to ASS and OTC expression and rhArg might be a potential therapy for TNBC.

MDA-MB-231 cell line was subsequently chosen as the model cell line for following experiments.

### Apoptosis induced by rhArg was not related to caspase 3 activation in MDA-MB-231 cells

**Figure 2:**
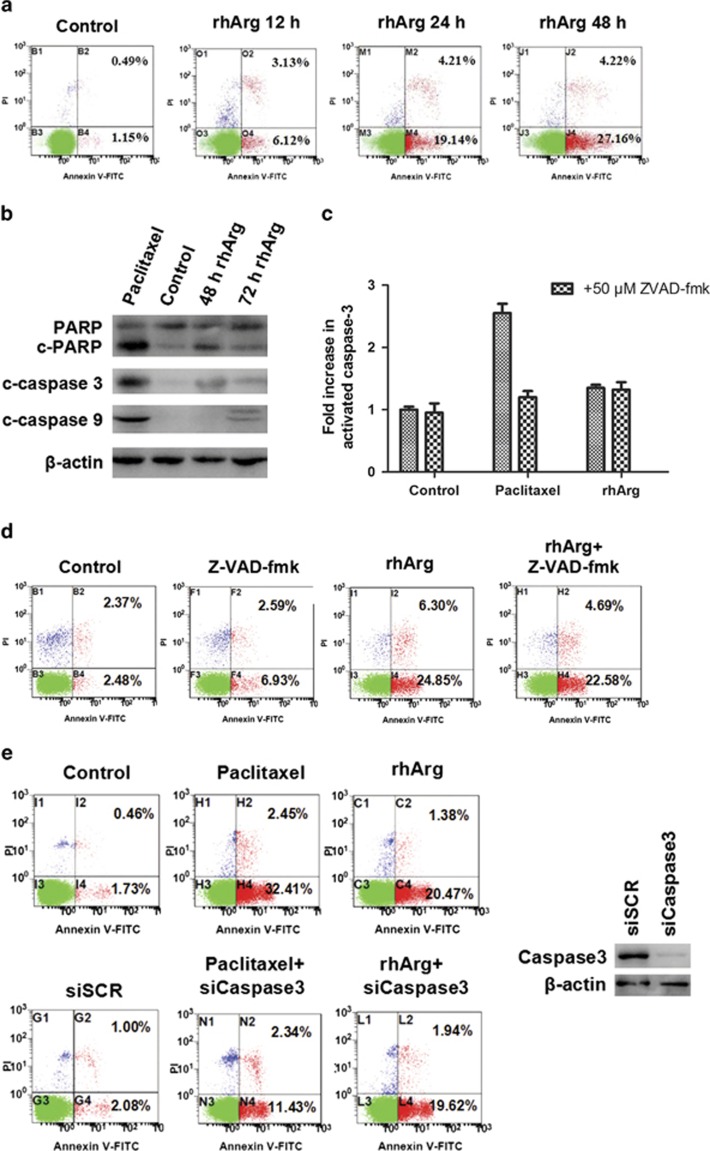
The role of caspase 3 in apoptosis induced by rhArg in MDA-MB-231 cells. (**a**) MDA-MB-231 cells were treated with 1 U/ml of rhArg for 48 h. The collected cells were incubated with Annexin V/PI, and then the samples were analyzed by flow cytometry. (**b**) MDA-MB-231 cells were incubated with 1 U/ml of rhArg for 24, 48 and 72 h. The protein levels of cleaved caspase 3, cleaved caspase 9 and PARP were detected by western blot analysis. Paclitaxel was used as positive control. (**c**) MDA-MB-231 cells were treated with vehicle (untreated), 50 nM paclitaxel for 24 h or 1 U/ml of rhArg for 48 h and 20 mM Z-VAD-fmk was used to pretreat these cells. After that, caspase 3 activity was measured by Fluorescence Assay Kit (Nanjing, Jiangsu Province, China) and values were normalized to vehicle. (**d**) MDA-MB-231 cells were incubated with 1 U/ml rhArg in the presence or absence of pretreatment of 20 mM Z-VAD-fmk for 48 h. The collected cells were incubated with Annexin V/PI, and then the samples were analyzed by flow cytometry. (**e**) MDA-MB-231 cells were transfected with siRNA twice targeting caspase 3. After 48 h transfection, cells were treated with or without arginase for 3 days. And then the samples were analyzed by flow cytometry. Paclitaxel was used as positive control

Most anticancer drugs induced cell death through apoptosis. We used Annexin V-FITC to determine whether rhArg could trigger apoptosis in TNBC MDA-MB-231 cells. As shown in Figure 2a, treatment with 1 U/ml of rhArg for 48 h induced obvious apoptosis of MDA-MB-231 cells when compared with control. In the classic form of apoptosis, apoptosis happens when caspases, particularly caspase 3, are activated. A little cleavage of caspase 9 and caspase 3, PARP could be observed after treatment of 1 U/ml of rhArg for 72 h (Figure 2b). Paclitaxel was used as a positive control. The quantitative analyses of the cleavage of caspase 3, PARP and caspase 9 were shown in Supplementary Figure 1. Next we used Z-VAD-fmk, an irreversible general caspase inhibitor, for further investigation. We examined the activation of caspase 3 by fluorescence assay. The activity of caspase 3 increased after MDA-MB-231 cells were treated with 1 U/ml of rhArg. But there was no significant difference in activated caspase 3 levels between rhArg combined with Z-VAD-fmk inhibitor group and rhArg alone group, whereas Z-VAD-fmk resulted in a significant reduction of activated caspase 3 levels in paclitaxel-treated MDA-MB-231 cells (Figure 2c). Meanwhile, Z-VAD-fmk showed few effect on the proportion of apoptosis induced by rhArg (Figure 2d). Furthermore, genetic approach, small interfering RNA (siRNA) targeting caspase 3, was employed. We observed that siCaspase 3 could not alter the cell apoptosis proportion induced by rhArg in MDA-MB-231 cells. Paclitaxel was used as a positive control (Figure 2e). Taken together, the results above showed that rhArg induced some cleavage of Caspase 3 and Caspase 9, but the cell death induced by rhArg might not be related to the caspase 3 cleavage.

### RhArg induced remarkable accumulation of autophagosomes in MDA-MB-231 cells

**Figure 3:**
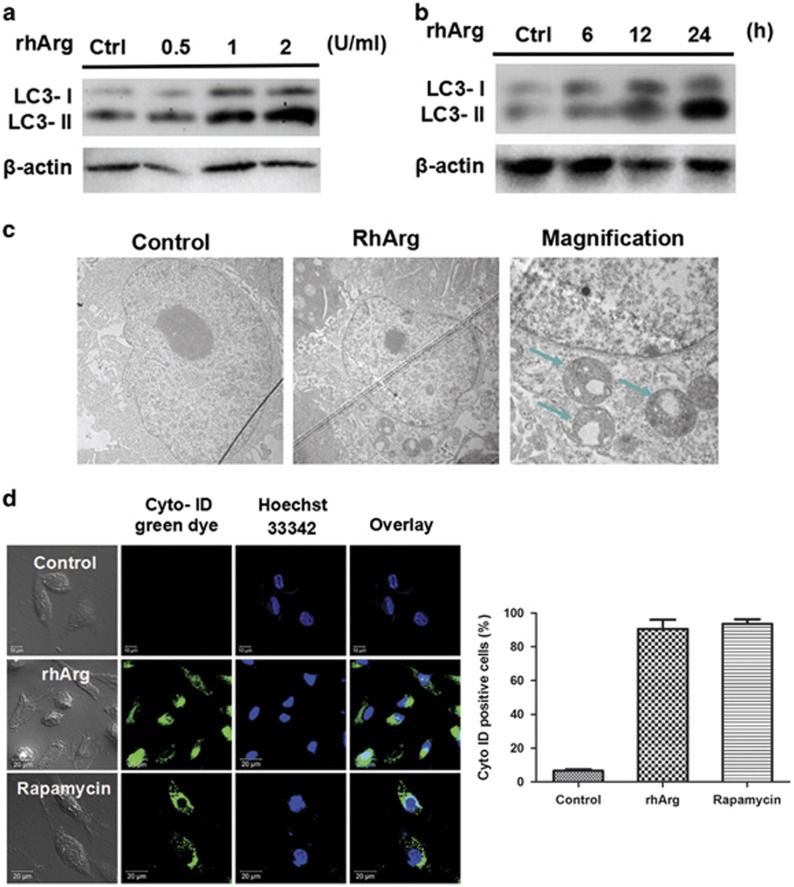
RhArg-induced autophagy in MDA-MB-231 cells. (**a**) MDA-MB-231 cells were treated with 0.5, 1 and 2 U/ml of rhArg for 24 h and the expression of LC3-I/II was measured by immunoblot analysis. (**b**) MDA-MB-231 cells were treated with 1 U/ml of rhArg for 6, 12 and 24 h and the expression of LC3-I/II was measured by immunoblot analysis. (**c**) Representative electron micrographs of MDA-MB-231 cells treated with vehicle control (50 nM rapamycin) or rhArg for 24 h were taken at × 5000 (left and middle) or × 20 000 (right). (**d**) MDA-MB-231 cells were treated with 1 U/ml of rhArg for 24 h. Rapamycin (50 nM) was regarded as a positive control. The green fluoresce was detected by confocal microscopy. Cyto-ID positive cells were counted manually by ImageJ (NIH, Bethesda, MD, USA) cell counter (*n*=3, means±S.D.)

Autophagy commonly occurs in human cancers and contributes to disease progression and chemotherapy resistance.^19, 20^ To determine whether rhArg induced autophagy in MDA-MB-231 cells, we measured the protein levels of LC3-I and LC3-II after exposure to rhArg. During autophagy, microtubule-associated protein 1 light chain 3 (LC3/Atg8) transforms from 18-kD LC3-I to 16-kD LC3-II, which translocates into autophagosome membrane. The amount of LC3-II is highly correlated with the extent of autophagic activity.^21^ Western blot analysis showed that rhArg induced a progressive conversion of cytoplasmic LC3-I to autophagosomic LC3-II in a dose- and time-dependent manner (Figures 3a and b). The quantitative analyses of LC3-II/*β*-actin were shown in Supplementary Figures 2 and 3.

To further confirm the induction of autophagy by rhArg, we verified the accumulation of autophagic vacuoles in MDA-MB-231 cells by transmission electron microscopy. Ultrastructural analysis showed that MDA-MB-231 cells treated with rhArg for 24 h appeared a crowd of crescent-shaped membranes and semispherical autophagic vacuoles compared with control (Figure 3c). Consistent with the results above, we demonstrated the formation of autophagic vesicles using Cyto-ID green dye, an autophagy detection stain, by laser confocal microscopy. Cyto-ID selectively labels preautophagosomes, autophagosomes and autophagolysosomes. Rapamycin, an inducer of autophagy, was used as a positive control. The appearance of green fluorescence indicated that both rhArg and rapamycin significantly induced autophagic response in MDA-MB-231 cells (Figure 3d).

All these results demonstrated that autophagy was induced by rhArg in MDA-MB-231 cells.

### Autophagic flux was triggered by rhArg in MDA-MB-231 cells

**Figure 4:**
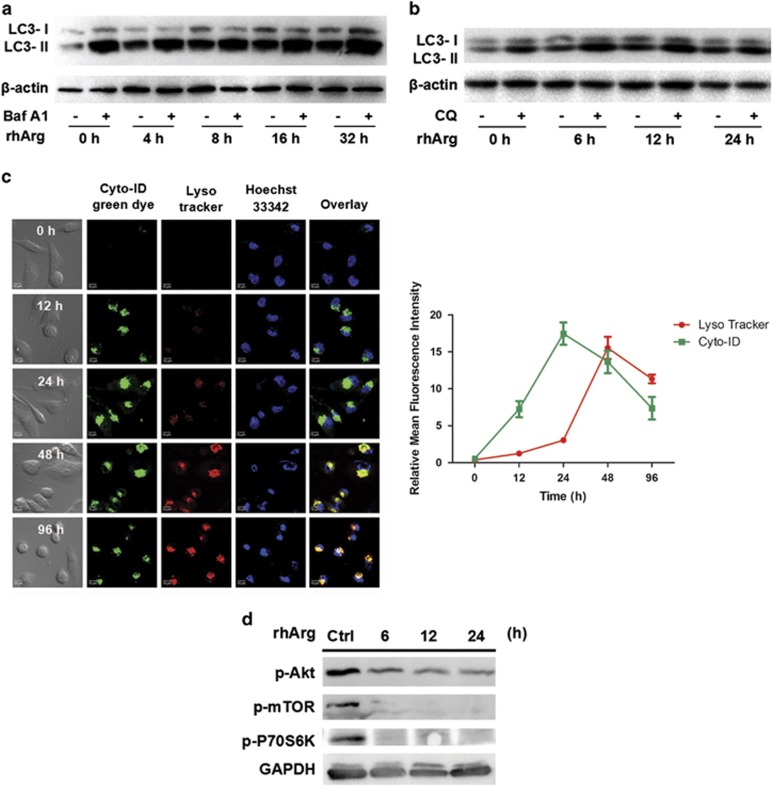
Autophagic flux measurements in MDA-MB-231 cells after rhArg treatment. (**a** and **b**) After exposure to rhArg for different time, MDA-MB-231 cells were treated with 2 U/ml rhArg in the presence Baf A1 (20 nM) and CQ (20 *μ*M) for another 4 h. Cell lysates were analyzed by immunoblot analysis. (**c**) Representative immunofluorescence images of MDA-MB-231 cells costained with Cyto-ID and LysoTracker Red after exposed to rhArg for the indicated times. green (Cyto-ID) and red (LysoTracker Red) dots in cells were counted using the ImageJ (*n*=3, means±S.D.). (**d**) MDA-MB-231 cells were treated with 1 U/ml rhArg for indicated times. Cell lysates were analyzed by immunoblot analysis

Nowadays, it has been believed that just analyzing the number of autophagic vacuoles is not an adequate method of measuring autophagic degradation activity (flux), because a growing number of autophagy-related structures can indicate increased generation and decreased clearance. One validated method to estimate autophagic flux is to carry out experiments in the absence or presence of lysosomal protease inhibitors (for example, leupeptin), lysosomal pH neutralizing agents (for example, chloroquine (CQ)) or drugs that specifically suppress the vacuolar type H^+^–ATPase complex (for example, bafilomycin A1).^22^ In our study, we treated MDA-MB-231 cells with rhArg for different times and applied bafilomycin A1 and CQ to inhibit LC3-II degradation at late stage. Compared with exposure to different times of rhArg alone, additional treatment with bafilomycin A1 caused an increased level of LC3-II, demonstrating that rhArg triggered an autophagic flux that results in lysosomal degradation of LC3-II (Figure 4a). The quantitative analyses of LC3 were shown in Supplementary Figure 4. As shown in Figure 4b and Supplementary Figure 5, the combination of CQ also elevated the expression of LC3-II in MDA-MB-231 cells compared with treatment of rhArg alone.

We also examined autophagy using Cyto-ID and Lyso-Tracker Red stains. LysoTracker Red DND-99, a fluorescent acidotropic probe, labels acidic organelles, such as lysosomes and autophagolysosomes. As Figure 4c showed that treatment of MDA-MB-231 cells with rhArg resulted in the occurrence of green fluorescent puncta in the cytoplasm within 12 h and massive accumulation reached a peak from 24 h, followed by a decrease in green dots afterwards. Simultaneously, red puncta continuously accumulated in the cytoplasm over time. In overlay images, yellow dots appeared at 48 h, indicating that autophagosomes transform into autophagolysosomes gradually.

Meanwhile, we investigated molecular pathways accompanying the induction of autophagy. Akt/mTOR signaling pathway is one of the crucial nutrient-sensing pathways, which can regulate autophagy in eukaryotic cells. Autophagy can be triggered when mTOR is inhibited by the diminished growth factors and amino acids during starvation. Western blot analysis showed that rhArg reduced Akt phosphorylation in MDA-MB-231 cells. This contributed to a decreased phosphorylation of mTOR, which is a significant downstream effector of Akt. Suppression of mTOR pathway dephosphorylated p70S6K, downstream target of mTOR along the treatment of rhArg in a time-dependent manner (Figure 4d). The quantitative analyses of p-Akt, p-mTOR and p-p70S6K were presented in Supplementary Figure 6.

Consequently, these results suggested that rhArg not only triggered the initial phase of autophagy, but also led to the maturation of autophagosomes to degradative autolysosomes.

### Blocking autophagy potentiated rhArg-triggered cytotoxicity in MDA-MB-231 cells

**Figure 5:**
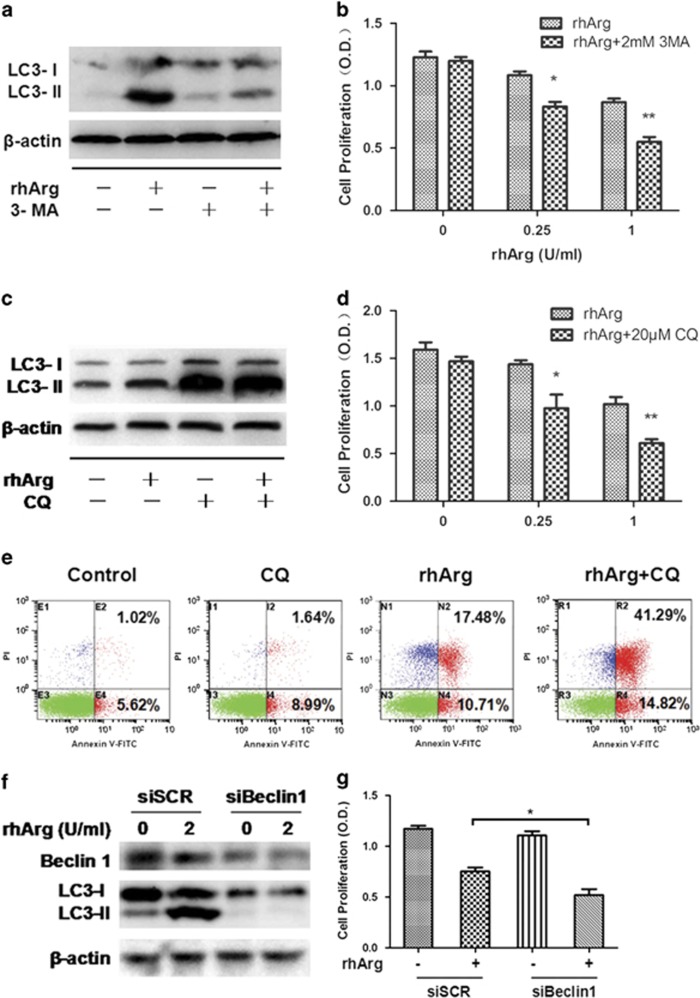
Suppressing autophagy accelerated rhArg-induced apoptosis and growth inhibition of MDA-MB-231 cells. (**a** and **c**) MDA-MB-231 cells were incubated with 1 U/ml rhArg for 24 h, in the presence or absence of CQ (20 *μ*M) and 3-MA (2 mM) and the expression of LC3-I/II was measured by western blot analysis. (**b** and **d**) MDA-MB-231 cells were incubated with rhArg for 72 h in the presence or absence of CQ (20 *μ*M) and 3-MA (2 mM), the cell viability was determined by MTT (*n*=3, means±S.D., *P<0.05, **P<0.01 *versus* each respective rhArg group). (**e**) MDA-MB-231 cells were incubated with 1 U/ml rhArg in the presence or absence of CQ (20 *μ*M) before flow cytometry analysis. (**f** and **g**) MDA-MB-231 cells were transiently transfected with Beclin1 siRNA before treatment of 1 U/ml of rhArg. (**f**) The levels of Beclin1 were detected by western blot analysis. (**g**) The viability after treatment was measured by MTT (*n*=3, means±S.D., *P<0.05 *versus* each respective rhArg group)

Previous studies revealed that autophagy could promote either cell survival or cell death relying on the type of stimuli, intracellular environment and apoptotic status.^23, 24, 25^ To determine the role of autophagy induced by rhArg, we exposed MDA-MB-231 cells to the combination of rhArg and autophagy inhibitors (CQ and 3-methyladenine (3-MA)). 3-MA inhibits autophagy by blocking autophagosome formation at early stage via the inhibition of class III phosphatidylinositol 3-kinases (PI-3K). As shown in Figure 5a and Supplementary Figure 7, the addition of 3-MA resulted in the decrease of LC3-II compared with rhArg alone. Meanwhile, inhibiting autophagy by 3-MA-enhanced rhArg-induced growth inhibition in MDA-MB-231 cells (Figure 5b). CQ suppresses the fusion of autophagosome and lysosome, which remarkably elevates the expression level of LC3-II. Western blot analyses revealed that 20 *μ*M of CQ elevated the expression level of LC3-II after rhArg treatment (Figure 5c). The quantitative analyses of LC3-II/*β*-actin were shown in Supplementary Figure 8. A significant increase in rhArg-induced cell growth inhibition was also observed after autophagy was inhibited by CQ (Figure 5d). Meanwhile, we found that CQ could enhance the growth inhibition induced by rhArg in HCC-1806, HCC-1937, BT-549 and HS-578T cells (Supplementary Figure 9). Consistently, we found that the addition of CQ augmented the proportion of apoptotic cells (56.11%) compared with treatment of rhArg alone (28.19%) (Figure 5e).

To rule out the possibility of off-target effect of autophagy inhibitors mentioned above, siRNA approach was employed to investigate the protecting role of rhArg-induced autophagy. Although the protein level of Beclin1, a key autophagy regulator, was downregulated, LC3-II was impaired in MDA-MB-231 cells treated by rhArg compared with scrambled control (SCR) siRNA transfection (Figure 5f). The quantitative analyses of Beclin 1 and LC3-II were presented in Supplementary Figure 10. Also, we observed that silence of Beclin 1 enhanced rhArg-induced cell growth inhibition compared with siSCR group (Figure 5g).

Collectively, the results showed that blocking autophagy enhanced rhArg-induced cytotoxicity of MDA-MB-231 cells.

### ROS was involved in rhArg-induced autophagy in MDA-MB-231 cells

**Figure 6:**
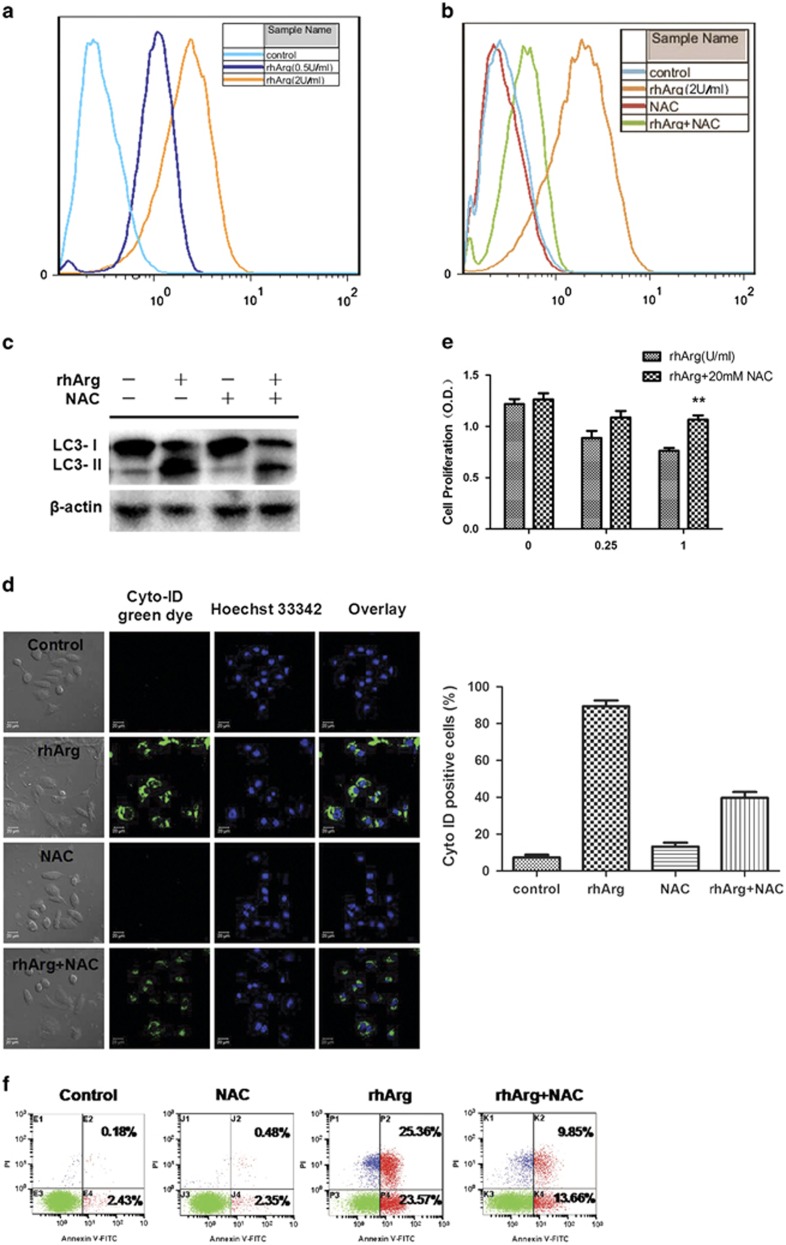
The role of ROS in rhArg-induced autophagy and apoptosis. (**a** and **b**) MDA-MB-231 cells were treated with 0.5 U/ml and 2 U/ml of rhArg with or without NAC (20 mM) for 3 days. Then, the treated cells were subjected to flow cytometry to measure ROS level. Data were processed by Flowjo (Tree Star, Inc., Ashland, KY, USA). (**c**) MDA-MB-231 cells were incubated with 1 U/ml of rhArg for 24 h in the presence or absence of NAC (20 mM) and the expression of LC3-I/II were measured by western blot analysis. (**d**) The green fluoresce was detected by confocal microscopy before rhArg in the presence or absence of NAC in MDA-MB-231 cells. Cyto-ID positive cells were counted manually by ImageJ cell counter (*n*=3, means±S.D.). (**e**) MDA-MB-231 cells were treated with rhArg in the presence or absence of NAC (20 mM) for 3 days. Then, the treated cells were subjected to MTT assay. (**f**) MDA-MB-231 cells were treated with 4 U/ml of rhArg in the presence or absence of NAC (20 mM) for 3 days and flow cytometry was used to detect Annexin/V apoptosis

Recently, mounting evidence has indicated that ROS accompanied the induction of autophagy in response to drug therapy in cancer cells.^26^ To determine whether ROS was involved in the treatment of rhArg, we used 2′,7′-dichlorodihydrofluorescein diacetate (DCFH-DA) probe to measure the production of ROS. In MDS-MB-231 cells, 0.5 U/ml and 2 U/ml of rhArg caused elevated ROS levels (Figure 6a). When *N*-acetyl-l-cysteine (NAC), a common antioxidant, was applied to minimize oxidative stress induced by 2 U/ml rhArg, the onset of total ROS generation was deterred (Figure 6b). To elucidate the underlying relationship between ROS and autophagy, we observed changes of LC3-II protein level induced by rhArg after pretreatment with NAC. As revealed in Figure 6c and Supplementary Figure 11, the rise of LC3-II conversion was attenuated by NAC notably, suggesting that NAC showed a considerable blocking effect of rhArg-induced autophagy. In parallel, we provided evidence that there was a decrease in Cyto-ID green fluorescence triggered by rhArg when MDA-MB-231 cells were pretreated with NAC (Figure 6d). To assess the role of ROS in rhArg-induced cell death, MTT assay and FITC-Annexin V/PI apoptosis assay were employed. Interestingly, NAC not only weakened the growth inhibition induced by rhArg (Figure 6e), but also rescued MDA-MB-231 cells from cell apoptosis to considerable extent (Figure 6f).

Taken together, ROS was highly involved in the autophagy and cytotoxicity induced by rhArg in MDA-MB-231 cells.

## Discussion

Recently, an increasing number of studies showed that arginine depletion exhibited great potential in malignancies therapy,^10, 27^ l-arginine, a precursor of polyamines, proline, glutamate creatine and cell-signaling molecule,^28^ has an essential role in several biological functions. In addition, arginine must be supplied in certain physiological conditions, such as tumor growth, because the requirement exceeds the production capacity.^29^ During a long time, refinements in the chemotherapy and antiangiogenic therapy regimens have led to some progress in the treatment of TNBC. However, the generation of successful therapies for TNBC has been less rewarding.^30, 31^ Therefore, the major challenge is to find more effective therapeutic regimens for TNBCs that are not responsive to endocrine therapy or trastuzumab. Taking into consideration of the promising results of rhArg, we endeavored to explore a novel therapy of TNBC by investigating cytotoxicity of arginine-depletion by rhArg.

It has been reported that the OTC and ASS, key enzymes in the synthesis of arginine from citrulline, were intimately linked with tumors' sensitivity toward rhArg.^27^ The key enzyme OTC and ASS of urea cycle could be regarded as an effective predictive marker for responsiveness to rhArg therapy, which facilitates pretreatment screening. In our study, ASS protein was not detectable in MDA-MB-231, HCC-1806, HCC-1937, HS-578T and BT-549 cells, rendering sensitivity to rhArg, whereas different levels of OTC proteins were observed in these TNBC. This might be the reason why these five breast cancer cells exibited different sensitivity to rhArg. Another arginine-depleting enzyme, arginine deiminase (ADI), has been evaluated in clinical phase II studies for malignant melanoma^32^ and HCC.^33^ There are some differences between rhArg and ADI. One among them is that the efficiency of ADI is highly correlated with ASS only, another essential enzyme in the synthesis of arginine from citrulline. Another difference is that ammonia is one of the product of ADI, whereas arginase converts arginine to ornithine and urea.^34^ Endogenously generated ammonia gas that acts like a free radical is very aggressive and cytotoxic until it is converted into ammonium ions.^35^

Here, we employed several methods to prove that autophagy was induced in TNBC MDA-MB-231 cells in response to rhArg treatment, including observing the formation of characteristic autophagosomes and the conversion of autophagic form of LC3. Meanwhile, we found that CQ and 3-MA, common autophagy inhibitors, led to remarkable increase of cytotoxicity in response to rhArg treatment in MDA-MB-231 cells. To exclude the possibility that CQ and 3-MA themselves may have functions other than inactivating autophagosomes, we adopted siRNA of Beclin1, which targets a component of the class III PI3 kinase complex that nucleates autophagosomes. Consistent with autophagy inhibitors' results, siBeclin1 potentiated the cell growth inhibition of MDA-MB-231 cells induced by rhArg. Taken together, our results indicated that the role of autophagy induced by rhArg was to protect breast cancer from cell death. To date, most studies showed that autophagy had a cytoprotective role in cancer therapy of ADI and rhArg.^8, 25, 36^

Nowadays, accumulating evidence suggests that ROS generation is important regulators for autophagy.^37, 38^ ROS are highly reactive oxygen free radicals or nonradical molecules that have essential roles in deciding cell fate. In consideration of this, it was of interest to study the role of ROS in autophagy and cell death. We found that NAC suppressed rhArg-triggered autophagy and rescued MDA-MB-231 cells from cell growth inhibition. However, it still needs further investigation, for recent data indicate that autophagy, apoptosis and even necrosis share some overlapping pathways through the induction of ROS.

In summary, we presented a novel promising therapy for TNBC and provided evidence for multitarget approaches of cancer therapy, in which rhArg was combined with autophagy-regulating drugs. Firstly, the rhArg-induced apoptosis in TNBC MDA-MB-231 cells was determined to be caspase 3 independent. Secondly, autophagic flux was induced by rhArg in MDA-MB-231 cells and inhibition of autophagy aggravated rhArg-induced cytotoxicity. Lastly, ROS had an essential role in the cytotoxicity of rhArg. Thus, this study provides new insights into the molecular mechanism of apoptosis and autophagy induced by rhArg in TNBC, suggesting a potential therapy for TNBC.

## Materials and Methods

### Cell culture

MDA-MB-231, BT-549, HCC-1806, HCC-1937 and HS-578T are characterized as triple-negative mammary carcinomas, which are clinically aggressive form associated with a poor prognosis. MDA-MB-231, BT-549 and HS-578T cells were cultured in DMEM medium (Gibco, CA, USA). HCC-1806 and HCC-1937 cells were cultured in 1640 medium (Gibco, CA, USA). The medium is supplemented with 10% bovine serum, 100 U/ml of penicillin and 100 *μ*g/ml streptomycin. The cells are cultured at 37 °C in a 5% CO_2_ incubator.

### Regents and antibodies

RhArg was expressed and purified as described previously.^13^ The specific activity of rhArg produced in *E. coli* BL21 expression system was 200 U/ml. The purity of rhArg is above 95%. One unit of rhArg was defined as the amount that converts 1 *μ*M of arginine to 1 *μ*M of ornithine per minute at 37 °C under the assay conditions. The activity of arginase was measured by QuantiChromTM Arginase Assay Kit, which was purchased from BioAssay Systems (Hayward, CA, USA).

3-(4,5-Dimetrylthiazol-2-yl)-2, 5-diphenyltetrazolium bromide (MTT), 3-MA, CQ and bafilomycin A1 were obtained from Sigma-Aldrich (St. Louis, MO, USA). Z-VAD-fmk, NAC and DCFH-DA were purchased from Beyotime Institute of Biotechnology (Haimen, Jiangsu Province, China). Caspase-3 Fluorescence Assay Kit was purchased from Keygen Biotech (Nanjing, China). Paclitaxel was obtained from Melonopharma (Dalian, Liaoning Province, China). Cyto-ID Green dye was purchased from ENZO Life Sciences, Inc. (Farmingdale, NY, USA). LysoTracker Red DND-99 was obtained from Invitrogen (Carlsbad, CA, USA). SiRNA was purchased from RiboBio Co., Ltd. (Guangzhou, China). X-tremeGENE siRNA transfection regent was from Roche (Basel, Switzerland). Fluorescein isothiocyanate (FITC)-AnnexinV/PI Apoptosis Detection kit was obtained from BD Bioscience (Franklin Lakes, NJ, USA). Antiactin was from Proteintech Group (Chicago, IL, USA). Anti-p70S6 Kinase Phospho (pS371) antibody was obtained from Epitomics (Burlingame, CA, USA). The secondary antibodies were purchased from MR Biotech (Shanghai, China). All the other antibodies were from Cell Signaling Technology (Danvers, MA, USA).

### MTT assay for cell proliferation assay

Cell proliferation was measured by MTT method, a colorimetric assay for measuring the activity of mitochondrial dehydrogenases, which reduce MTT to formazan. Cells were plated in 96-well plates at a density of 5 × 10^3^ cells/100 *μ*l. After treatment,cells were incubated with MTT reagent (0.5 mg/ml) for 4 h at 37 °C. After removing the supernatant, formazan crystal was dissolved in DMSO and the absorbance was measured at 570 nm.

### Apoptosis assay

To measure early/late apoptotic or necrotic cell death, cells were detected by Calibur flow cytometry (Becton-Dickinson, Fullerton, CA, USA) with Annexin V-FITC/PI Apoptosis Detection Kit (BD Biosciences, San Diego, CA, USA). Briefly, MDA-MB-231 cells were harvested after treatment with rhArg, and then the cells were treated with Annexin V-FITC/PI for at least 15 min in the dark according to the manufacturer's instructions.

### Immunoblot analysis

Breast cancer cells were collected for analysis of protein expression levels. After treated with rhArg, cells were harvested in 0.01 M phosphate-buffered saline (PBS, pH 7.4) and re-suspended in cell lysis buffer (Beyotime Biotechnology, Haimen, Jiangsu Province, China) for 30 min on ice. Equivalent amount of protein was loaded into sodium dodecyl sulfate polyacrylamide gel electrophoresis and transferred onto a polyvinylidene difluoride membrane. Next, the membranes were blocked with 5% skim milk in tris-buffered saline with Tween 20 (TBST) and then probed with primary antibodies and peroxidase-conjugated secondary antibodies. Subsequently, the membranes were visualized with an enhanced chemiluminescent detection kit (Pierce, Rockford, IL, USA).

### Caspase-3 fluorescence assay kit

MDA-MB-231 cells were seeded in 6-well plates and treated with 100 nmol/l paclitaxel or 1 U/ml rhArg. These groups were compared with cells treated with additional 20 mM Z-VAD-fmk. After that, the caspase-3 activity was assayed according to the manufacturer's instructions.

### Transmission electron microscopy

MDA-MB-231 cells were fixed in a solution containing 2% glutaraldehyde in 0.1 M PBS (pH 7.4) for 2 h. And then the samples were washed extensively with 0.1% buffered osmium tetroxide. Subsequently, cell samples were fixed in 0.1 M cacodylate buffer including 0.1% CaCl_2_ for at least 30 min and then dehydrated in increasing concentrations of ethanol and polymerized at 60 °C for 2 days. After being cut, the sections were stained with uranyl acetate and lead citrate. Next, the samples were stained with uranyl acetate and lead citrate, and then they were examined by a JEM 1230 transmission electron microscope (JEOL, Tokyo, Japan) at a voltage of 60 kV.

### SiRNA transfection

SiRNA targeting Beclin-1 (sense sequence: 5′-CAGUUUGGCACAAUCAAUA-3′ antisense sequence: 5′-GUCAAACCGUGUUAGUUAU-3′), siRNA targeting caspase 3 (sense sequence: 5′-AGUGAAGCAAAUCAGAAAC-3′ antisense sequence: 5′-UCACUUCGUUUAGUCUUUG-3′) and negative control siRNA were obtained from Guangzhou RiboBio Co., Ltd (Guangzhou, China). Cells were transfected with siRNA by X-tremeGENE siRNA transfection regent according to the manufacturer's instructions. After 48 h of transfection, MDA-MB-231 cells were treated with 1 U/ml of rhArg for 24 h. We investigated the efficiency of siRNA-mediated Beclin-1 silence by western blot. The viability after treatment was measured by MTT.

### Fluorescent staining

After treated with rhArg at indicated concentrations for 24 h, MDA-MB-231 cells were stained with Cyto-ID Autophagy Detection Kit and LysoTracker Red DND-99 according to the manufacturer's instruction. Rapamycin, an autophagy inducer, served as a positive control. After treated with Cyto-ID Green dye, Hoechst 33342 and LysoTracker Red DND-99 at 37 °C for 30 min, MDA-MB-231 cells were detected by laser confocal microscopy immediately.

### Measurement of intracellular ROS

Production of intracellular ROS was detected by fluorescent dye DCFH-DA. After treating with rhArg in the presence or absence of NAC (20 mM), cells were incubated with DCFH-DA (5 mM) for 30 min at 37 °C and then washed with serum-free medium for three times. Fluorescence intensity of cells was measured by flow cytometry at excitation wavelength of 488 nm and emission wavelength of 525 nm.

### Statistical analysis

GraphPad Prism 5 was used to carry out statistics analysis. Data were presented as the means±S.D. using Student's *t*-test (two-tailed) or one-way analysis of variance for analyzing the difference between groups. Levels of *P*<0.05 were considered statistically significant.

## Abbreviations

ADI: arginine deiminase
ASS: sucargininosuccinate synthetase
CQ: chloroquine
DCFH-DA: 7′-dichlorodihydrofluorescein diacetate
HCC: hepatocellular carcinoma
HER2: human epidermal growth factor receptor type 2
MTT: 3-(4, 5-dimetrylthiazol-2-yl)-2, 5-diphenyltetrazolium bromide
NAC: *N*-acetyl-l-cysteine
OTC: ornithine transcarbamylase
rhArg: recombinant human arginase
ROS: reactive oxygen species
TNBC: triple-negative breast cancers
3-MA: 3-methyladenine
