# Supplementary material for: Blocking autophagy enhanced cytotoxicity induced by recombinant human arginase in triple-negative breast cancer cells
Source: Cell Death Dis. 2014 Dec 11;5(12):e1563–. doi: 10.1038/cddis.2014.503 (PMC4454157; doi:10.1038/cddis.2014.503)
Supplement: Supplementary Figures [file cddis2014503x1.doc]

**Figure S1**

**
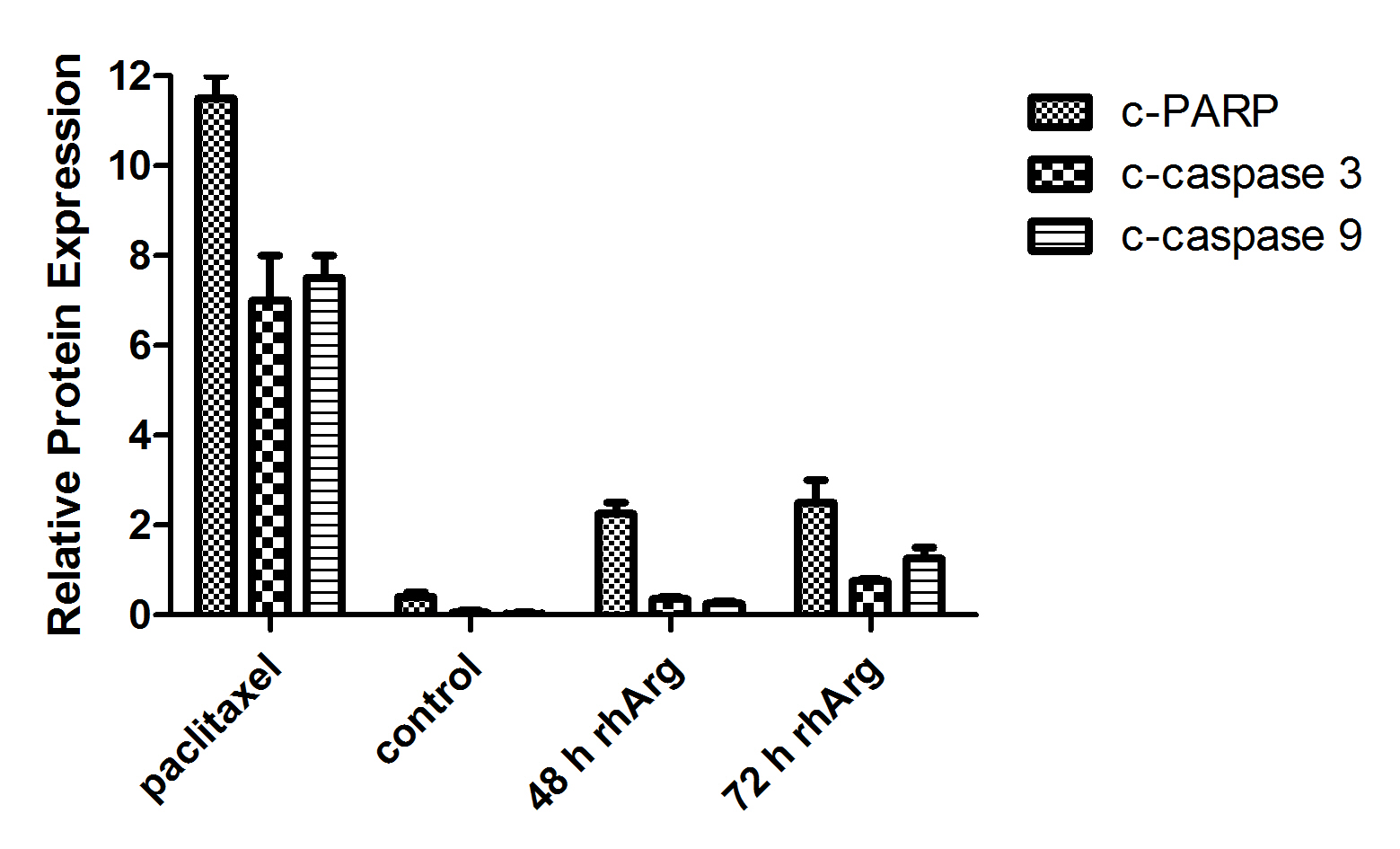
**

The relative protein expressions of c-PARP, c-caspase 3 and c-caspase 9 were qualified by ImageJ software. (N =3, mean ± SD)

**Figure S2**

**
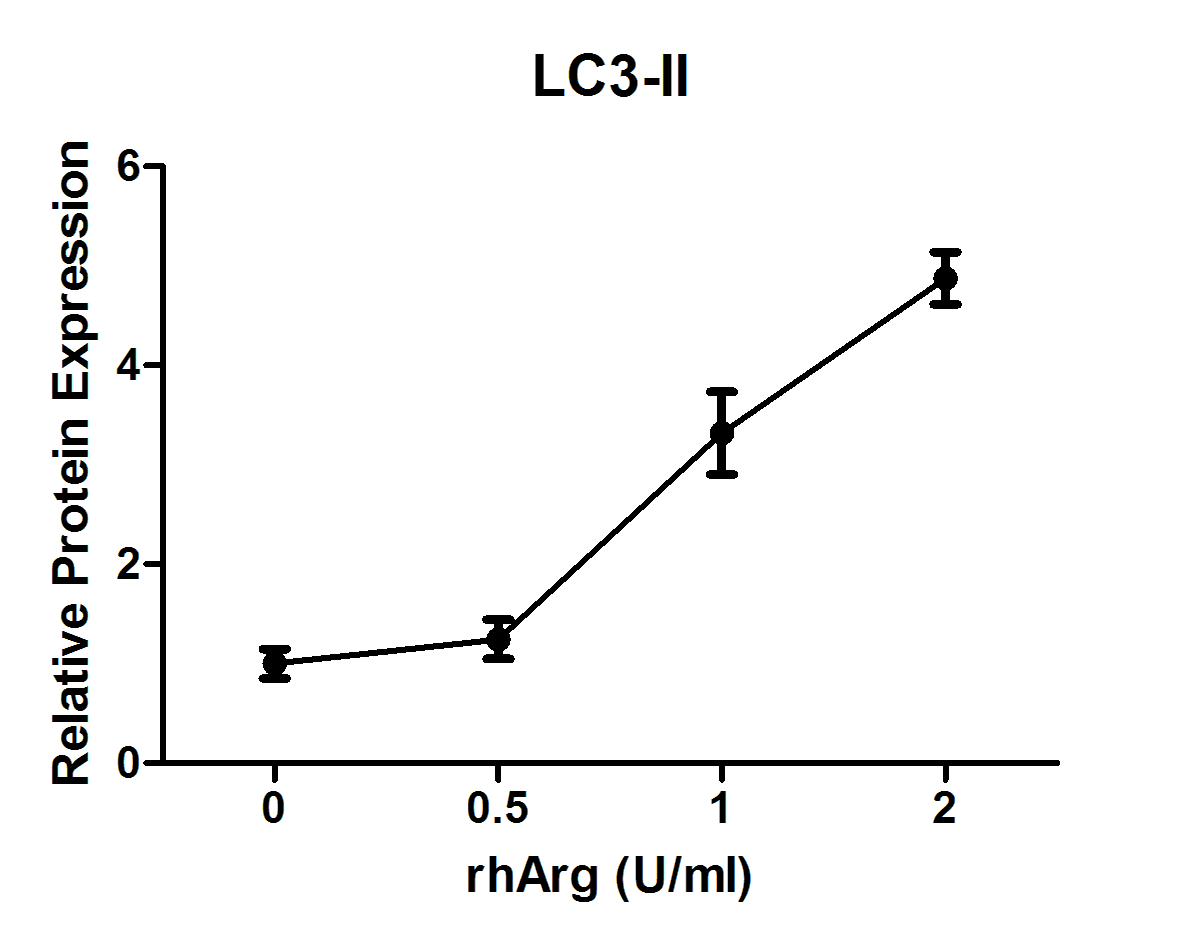
**

**LC3-II/β-actin**

The relative protein expression of LC3-II was qualified by ImageJ software. (N =3, mean ± SD)

**Figure S3**

**
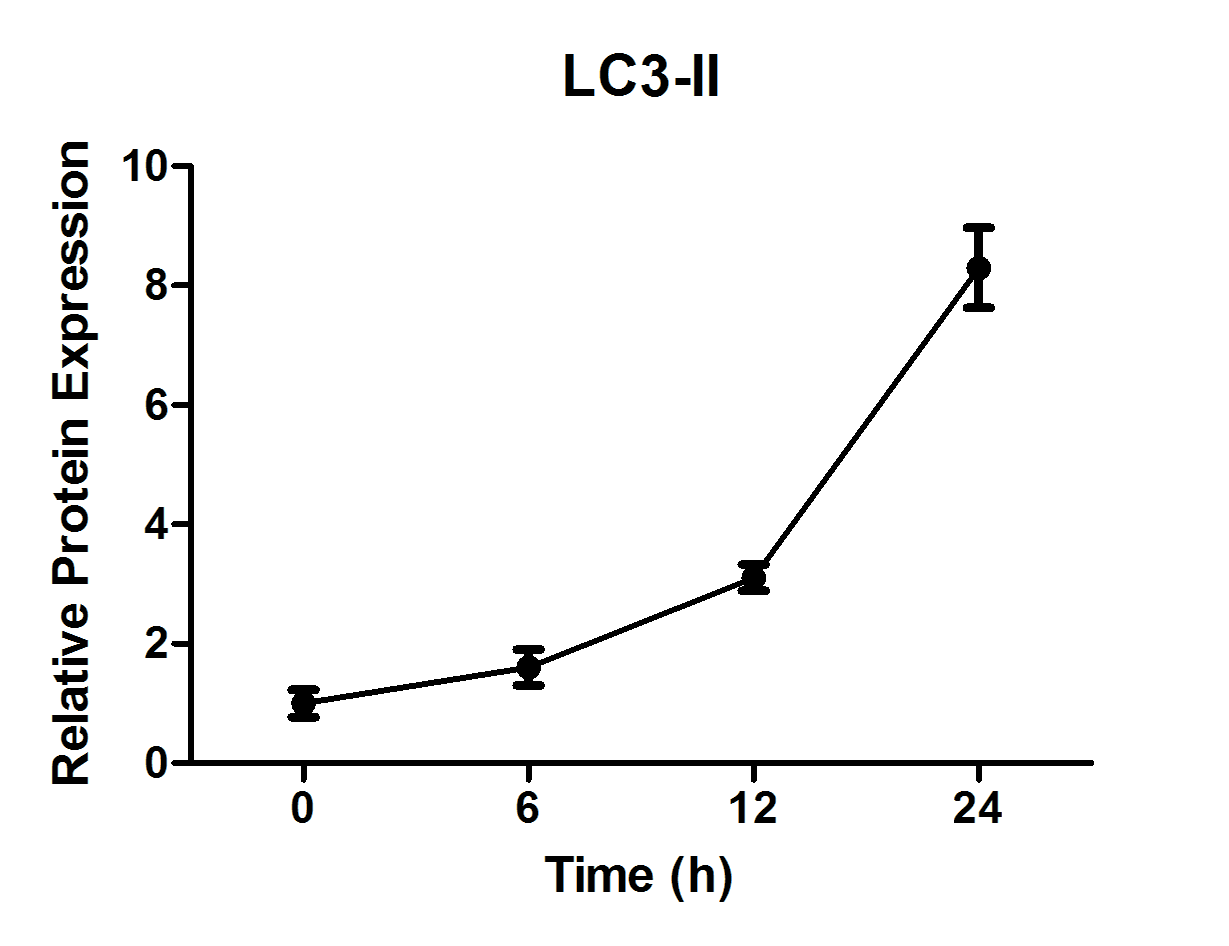
**

**LC3-II/β-actin**

The relative protein expression of LC3-II was qualified by ImageJ software. (N =3, mean ± SD)

**
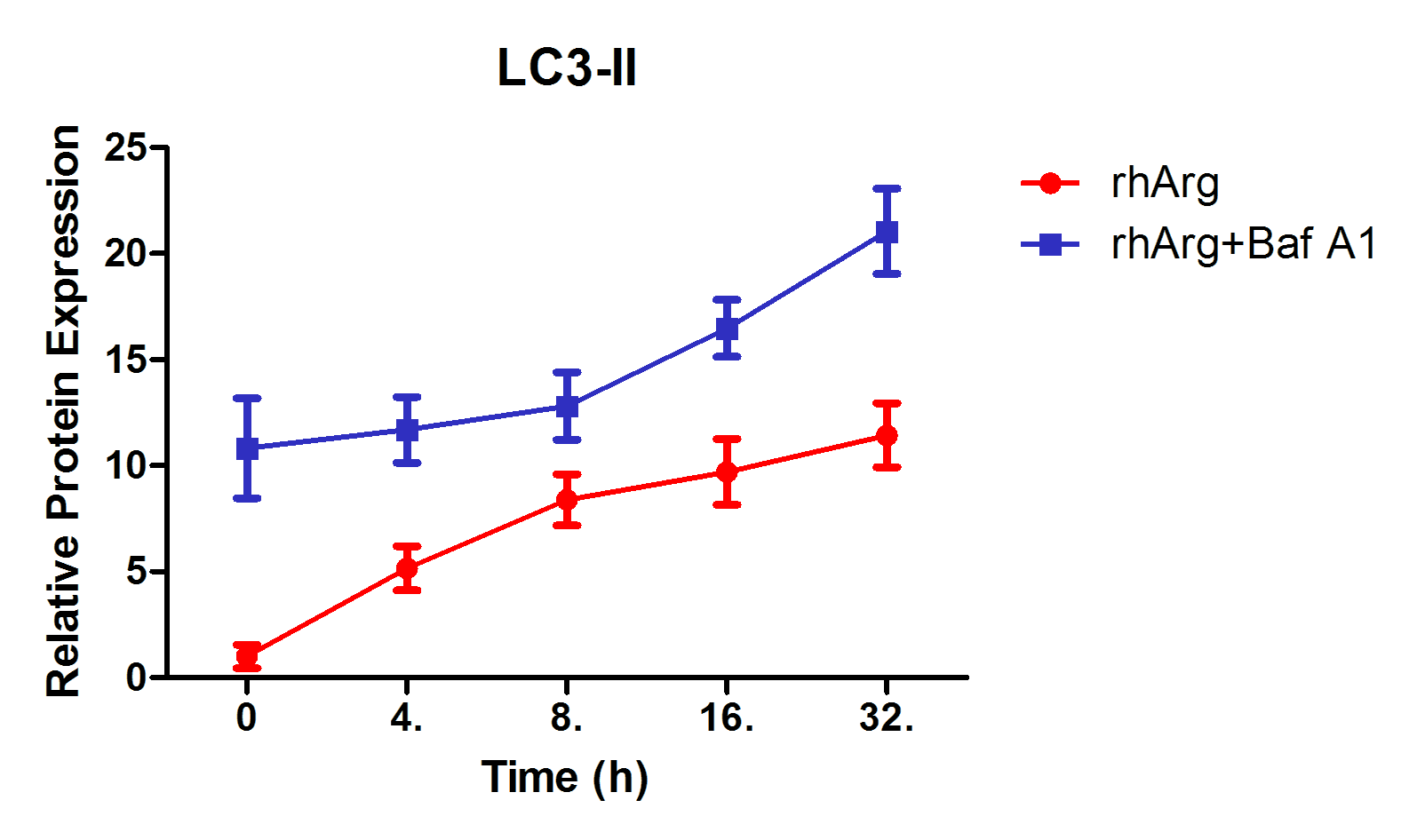
Figure S4**

**LC3-II/β-actin**

The relative protein expression of LC3-II was qualified by ImageJ software. (N =3, mean ± SD)

**Figure S5**

**
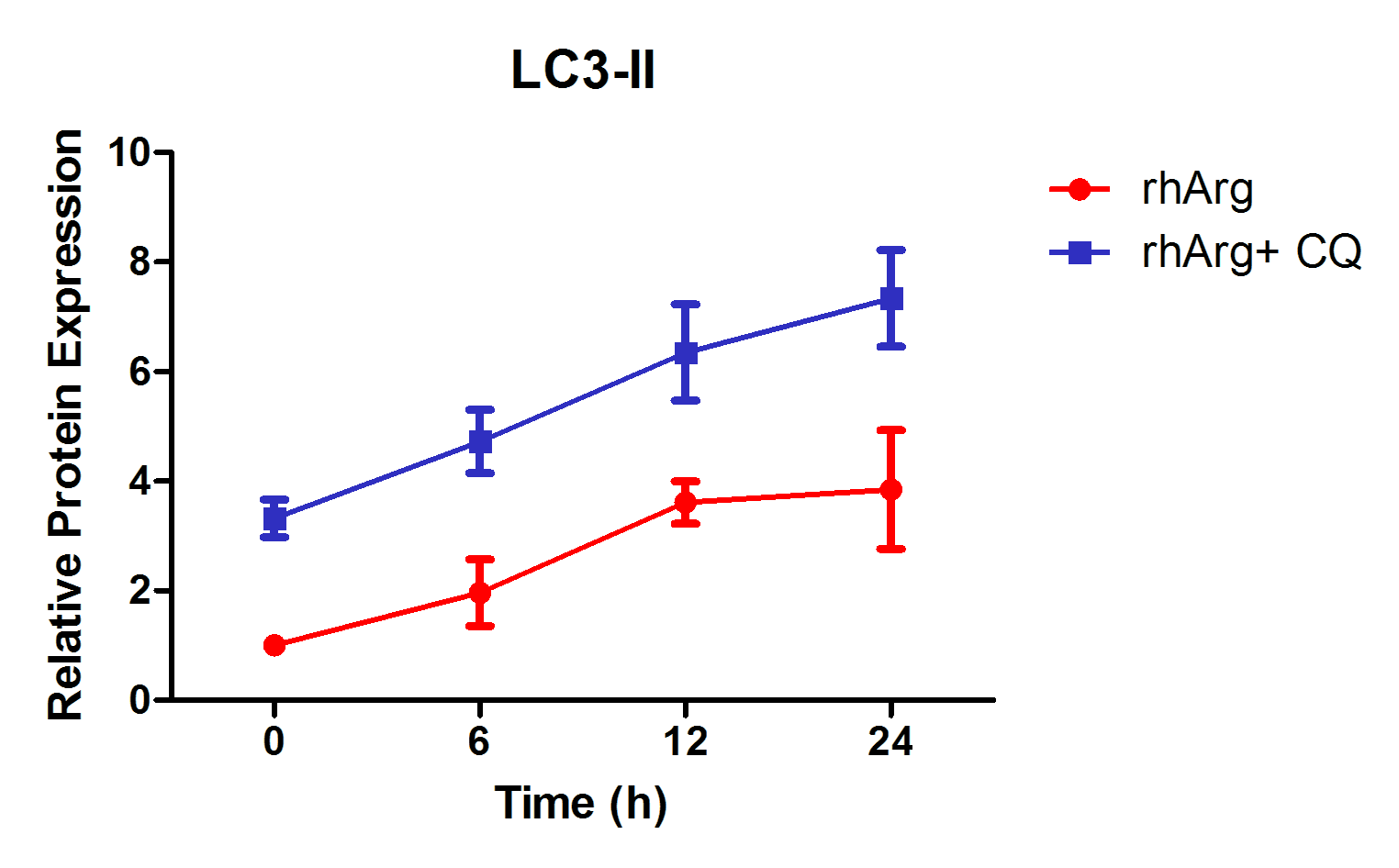
**

**LC3-II/β-actin**

The relative protein expression of LC3-II was qualified by ImageJ software. (N =3, mean ± SD)

**Figure S6**


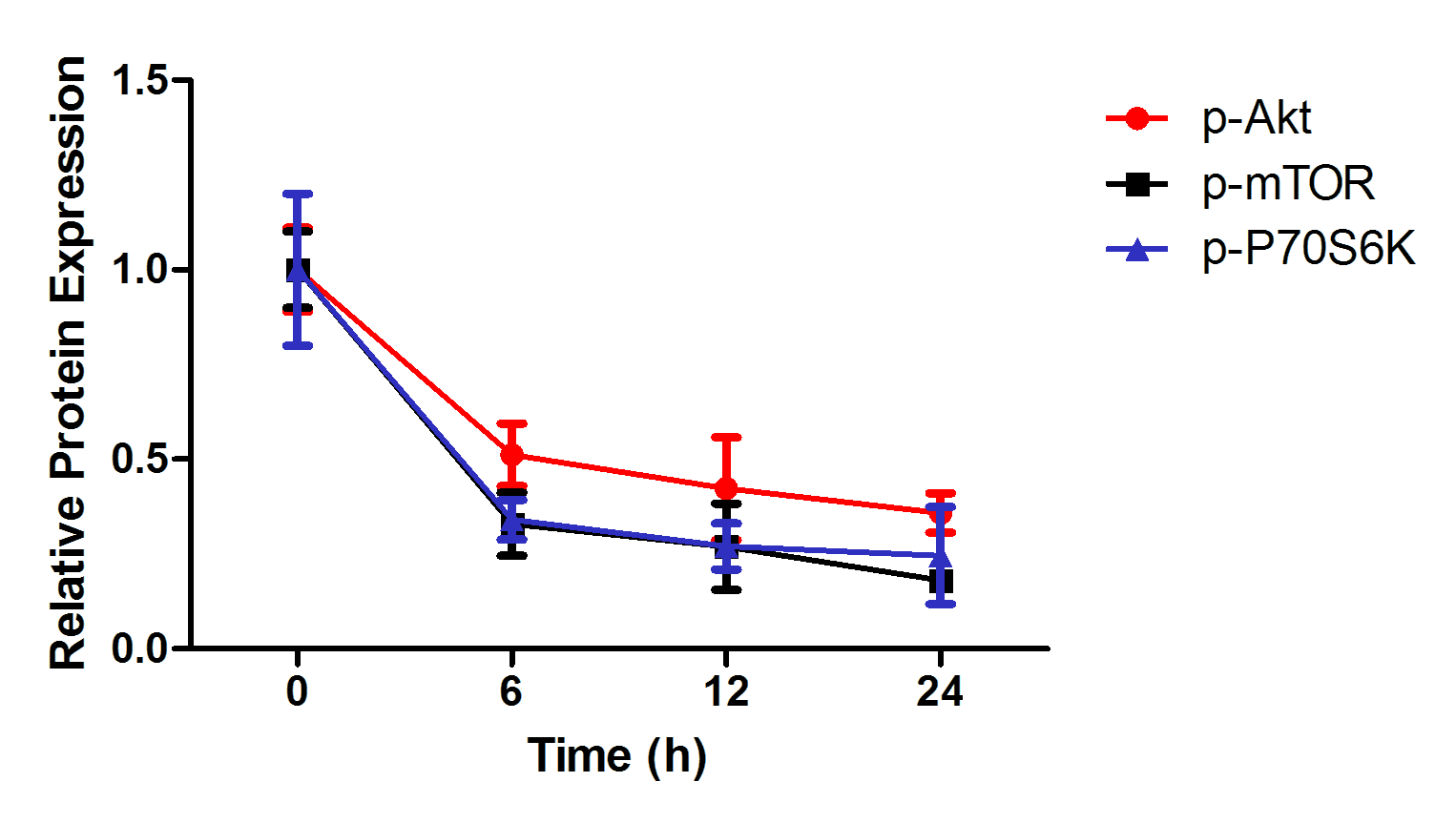


The relative protein expression of p-Akt, p-mTOR, p-P70S6K were qualified by ImageJ software. (N =3, mean ± SD)

**
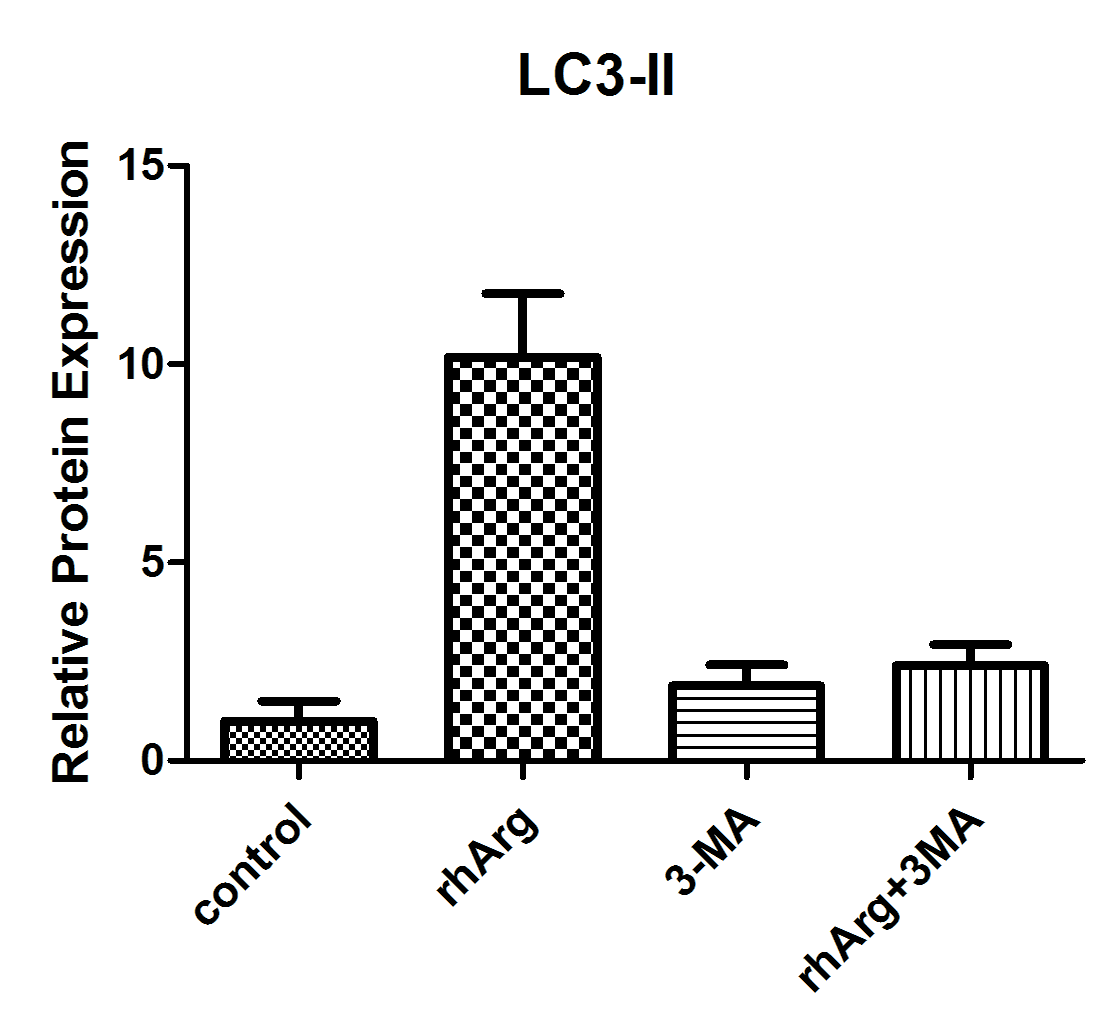
Figure S7**

**LC3-II/β-actin**

The relative protein expression of LC3-II was qualified by ImageJ software. (N =3, mean ± SD)

**
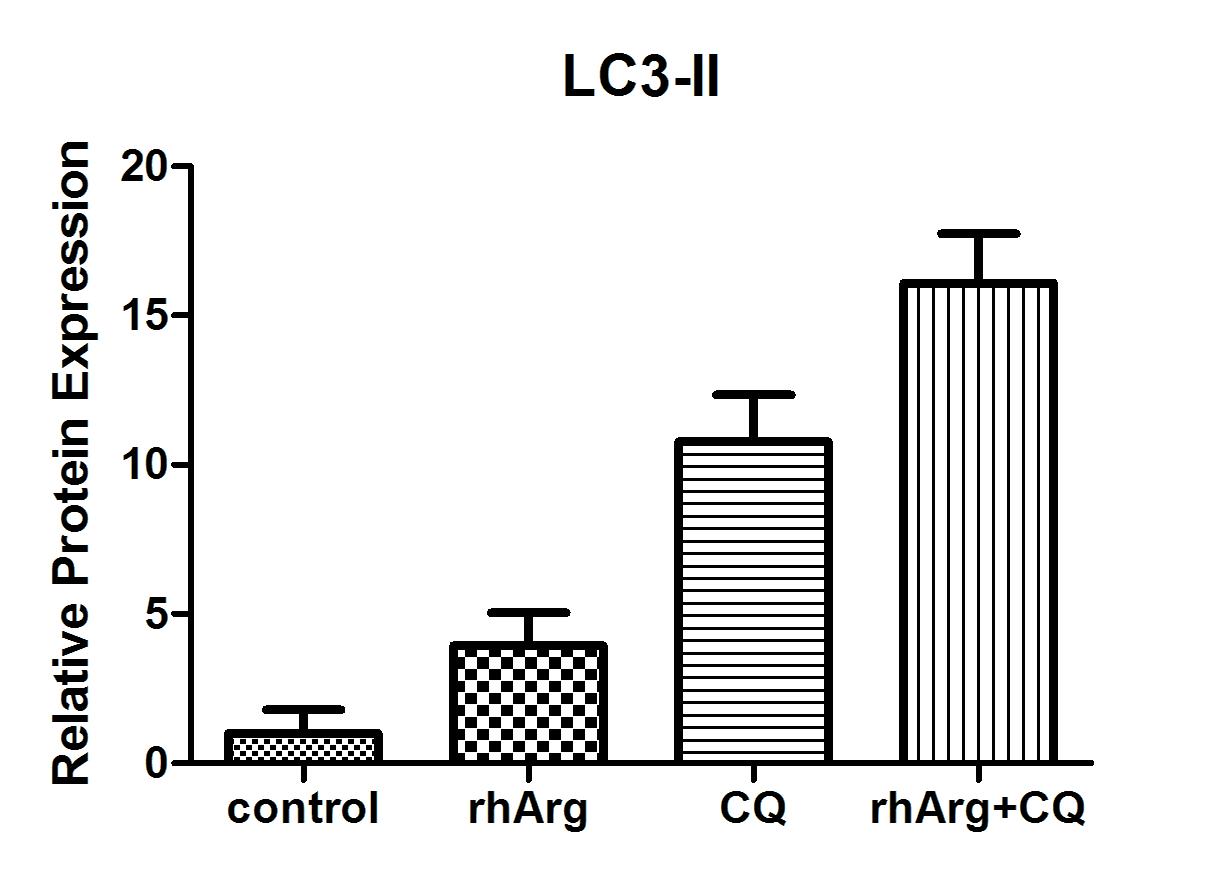
Figure S8**

**LC3-II/β-actin**

The relative protein expression of LC3-II was qualified by ImageJ software. (N =3, mean ± SD)


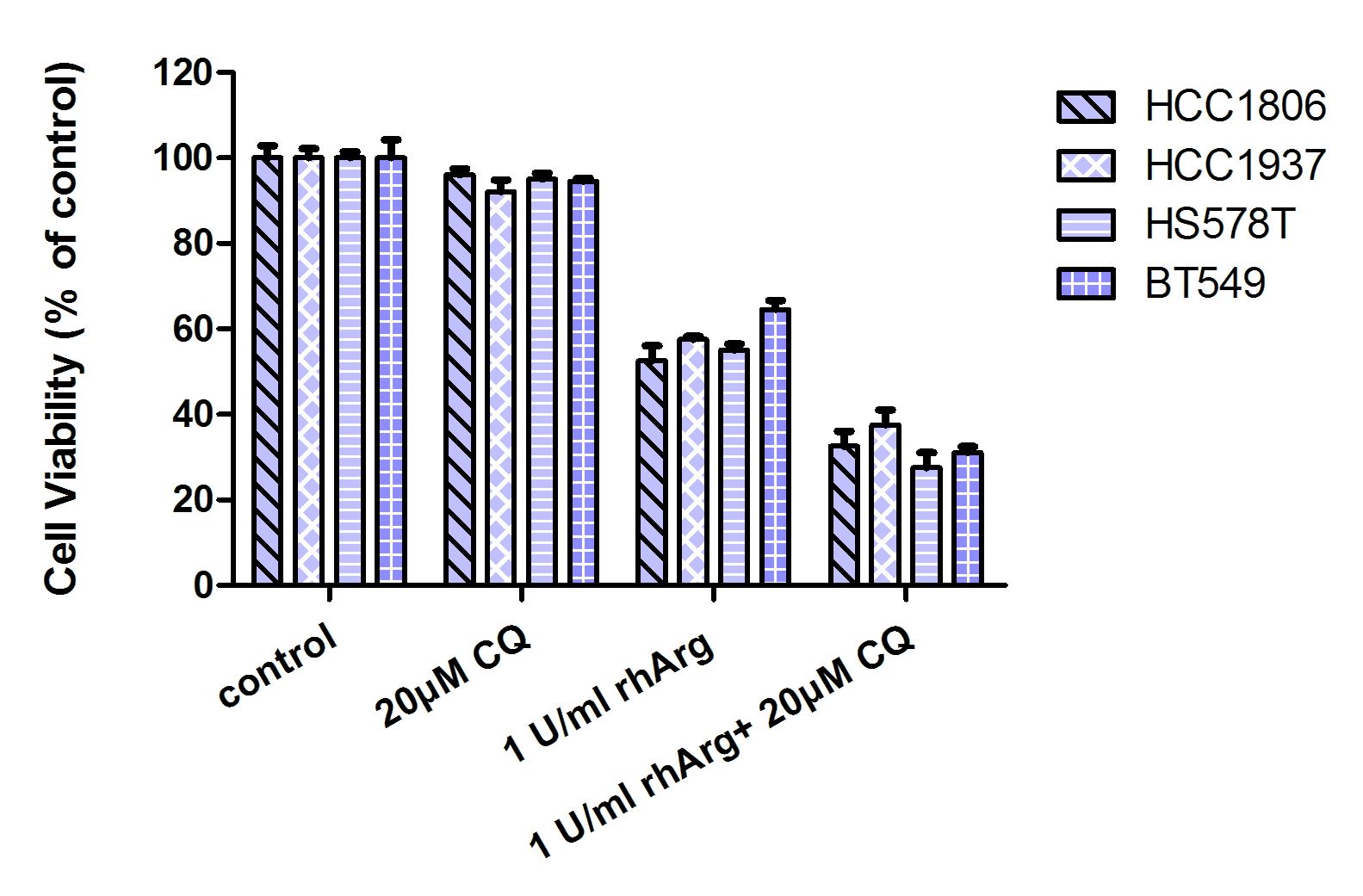
**Figure S9**

HCC1806, HCC1937, HS578T and BT549 cells were incubated with rhArg for 72 h in the presence or absence of CQ (20 μM), the cell viability was determined by MTT (n=3, means±SD).

**
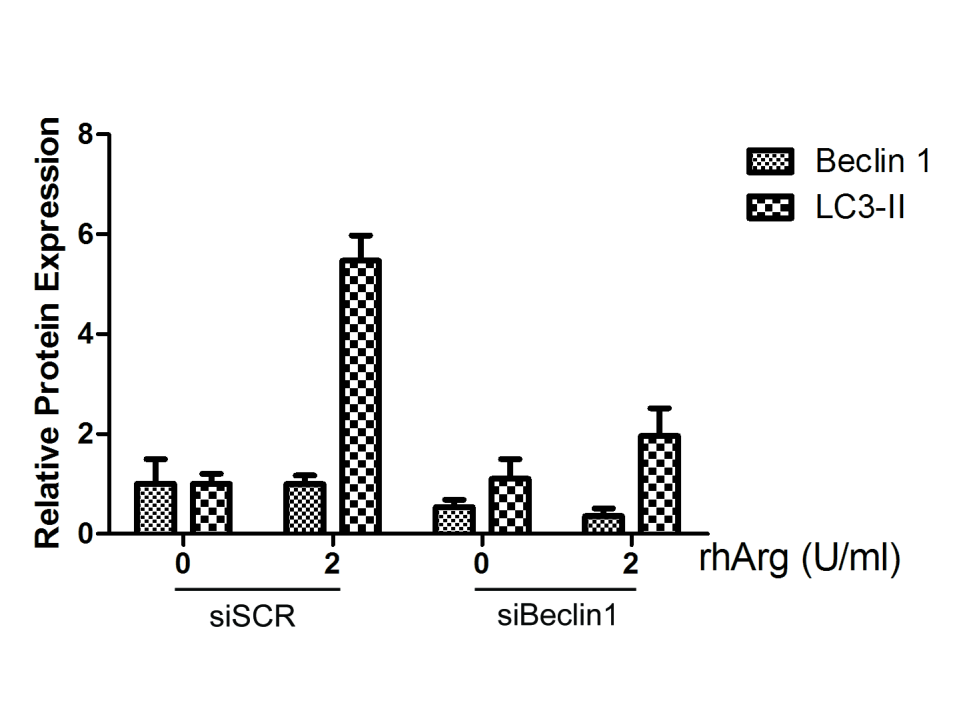
**

**Figure S10**

The relative protein expression of LC3-II and Beclin 1 were qualified by ImageJ software. (N =3, mean ± SD)

**Figure S11**

**
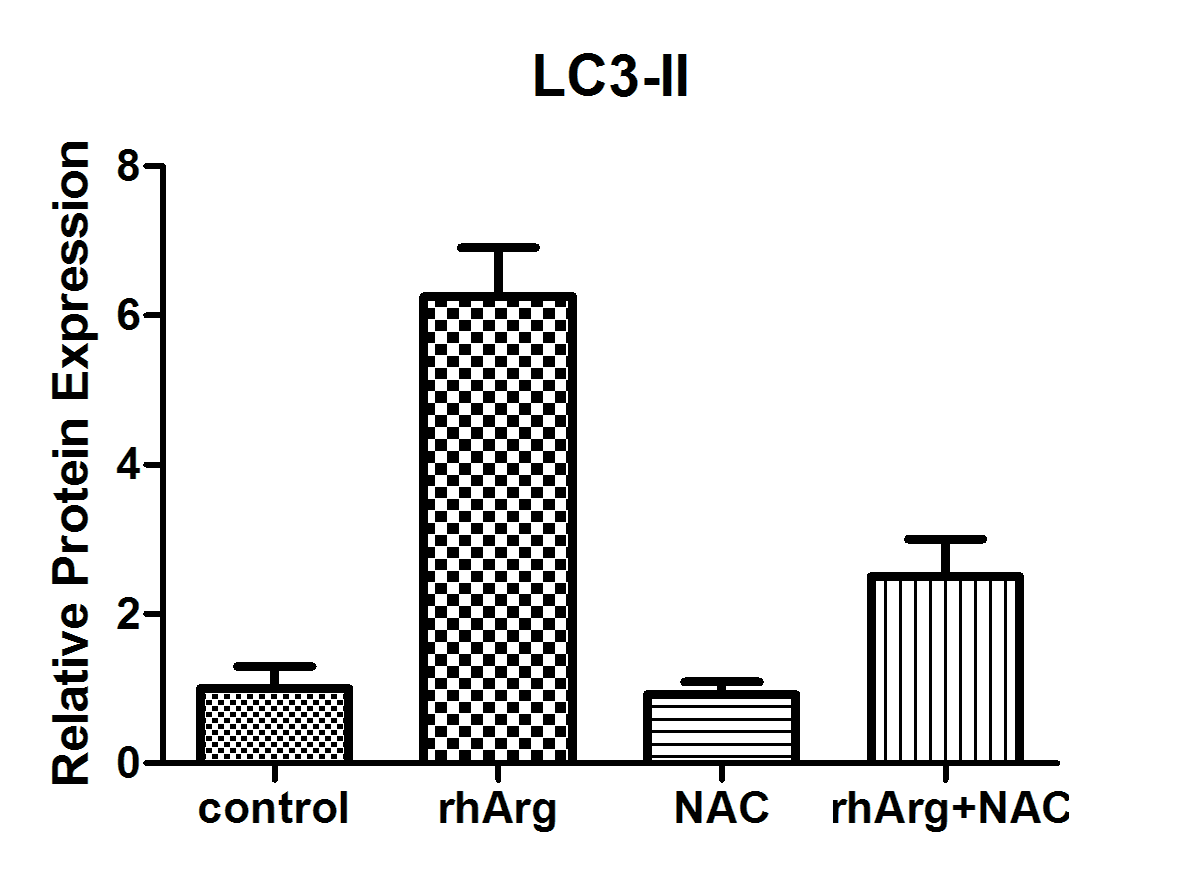
**

**LC3-II/β-actin**

The relative protein expression of LC3-II was qualified by ImageJ software. (N =3, mean ± SD)
